# The biological and evolutionary consequences of competition between DNA sequences that benefit the cell and DNA sequences that benefit themselves

**DOI:** 10.1093/nar/gkaf589

**Published:** 2025-07-08

**Authors:** Arnold J Bendich, Scott O Rogers

**Affiliations:** Department of Biology, University of Washington, Seattle, WA 98195, United States; Department of Biological Sciences, Bowling Green State University, Bowling Green, OH 43403, United States

## Abstract

Mobile genetic elements (e.g. transposons and introns) are genomic parasites, DNA sequences that can proliferate within host genomes. In prokaryotes, these elements are minimized by purifying selection and host-cell defenses. In eukaryotes, however, these parasitic sequences may comprise most of the genome. This extreme imbalance in eukaryotes is maintained by a metabolically quiescent germline through which the genomic parasites are transmitted to the next generation via gametes and spread throughout the population via sexual reproduction. Some eukaryotes avoid the threat posed by genomic parasites by eliminating them from somatic cells, whereas others retain the parasites in somatic cells and attempt to suppress them by epigenetic silencing mechanisms. Here, we review the evidence for the ongoing competition between host and parasite for genome occupancy. We conclude that defenses against mobile genetic elements vary greatly among organisms, and this variation accounts for the enormous range in genome size among organisms.

## Graphical Abstract

**Graphical Abstract:**
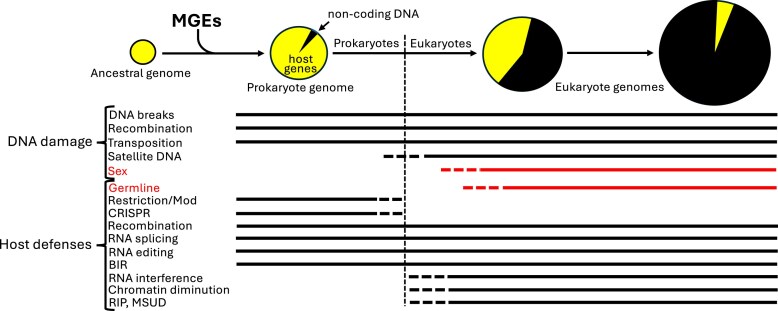

## Introduction

At the most basic level, two DNA sequence categories exist: those that contribute to survival of the organism (e.g. genes) and those that contribute primarily to their own survival. The latter, termed parasitic (or “selfish”) DNA, include viruses, transposons, and introns, collectively called mobile genetic elements (MGEs). These two categories are in constant competition. MGEs introduce mutations into the genome, and cells have developed mechanisms to suppress the effects of MGEs. In terms of genome fraction, the first category has the upper hand in prokaryotic organisms (Bacteria and Archaea), as genes and their regulatory DNA sequences account for almost the entire genome. Gene products that defend against MGEs and sequences between genes typically account for ∼1% of the genome in free-living species [1–3] (see Supplementary Table S1 and Supplementary Fig. S1). In eukaryotes, defenses against MGEs are weak, allowing the balance to be reversed. Parasitic DNA and its descendant sequences can account for most of the nuclear genome in some animals and plants, with large differences among related organisms [4, 5]. Similarly, large and variable fractions of “repetitive” (parasitic) sequences were reported for mitochondrial and plastid genomes [6–8]. How can we explain why such variation exists in eukaryotic genomes but not prokaryotic genomes?

We contend that the competition between DNA sequences that promote the survival of the organism and those that prioritize their own propagation plays a dominant role in shaping genetics, biology, and evolution.

## A brief overview of MGEs

MGEs fall into several categories. Viruses replicate within a host cell and spread to other cells through an extracellular path. Transposons and introns are MGEs that lack the protective covering of viruses; they move or transfer a copy to other parts of the host genome. Some also spread to new cells through cellular connections by, for example, bacterial plasmids or by the fusion of eukaryotic gametes during sexual reproduction.

MGEs can move within a genome, and some can rapidly increase their copy number. They insert at sites of DNA breakage, creating direct repeats (DRs) surrounding the inserted element. McClintock [9] identified transposable elements as a two-component system in maize: the autonomous Ac (activator) and the nonautonomous Ds (dissociation). Ds elements were later shown to comprise Ac derivatives lacking the transposase gene and in some cases all Ac sequences except for short repeats at the Ac termini [10]. The DNA-to-DNA movements of high-copy Ds and low-copy Ac elements require the transposase to find a sequence-specific site for insertion. Since DNA strands must be cut to allow entry of this (and any other) mobile element, completion of the insertion event must involve the sealing of the cut sites, an activity supplied by the host cell. The concept of a transposase as a requirement for site-specific entry became a hallmark for Type II transposable elements (such as Ac/Ds) and later for Type I transposable elements that move via an RNA intermediate. Type I, RNA-based elements are commonly found in much higher copy number per genome than Type II elements: their multi-copy transcription intermediate, a SINE (short interspersed nuclear element), is present in about a million “copies” in the human genome [11]. For these transposable elements, repair of the insertion site creates single-strand gaps that become DRs at the boundaries of the element after repair completes the insertion process [12]. The DRs are a hallmark of all adequately characterized MGEs; they are left behind in tandem even after the MGE exits its original insertion site.

While many transposon types rely on transposases for movement, the enormous size of the nuclear genome is largely attributable to MGEs that move without a dedicated transposase. A DNA sequence need not require its own sequence-specific transposase to move within a genome. For example, MITEs (miniature inverted-repeat transposable elements) are Type II transposable elements that carry no transposase sequence and are thus nonautonomous. They utilize transposases from other transposons, not identified as MITEs, that insert at specific target sites [13]. MITEs are found in many eukaryotes, although at relatively low copy number and, along with other Type II elements, they do not contribute greatly to the size of nuclear genomes.

**Figure 1.**
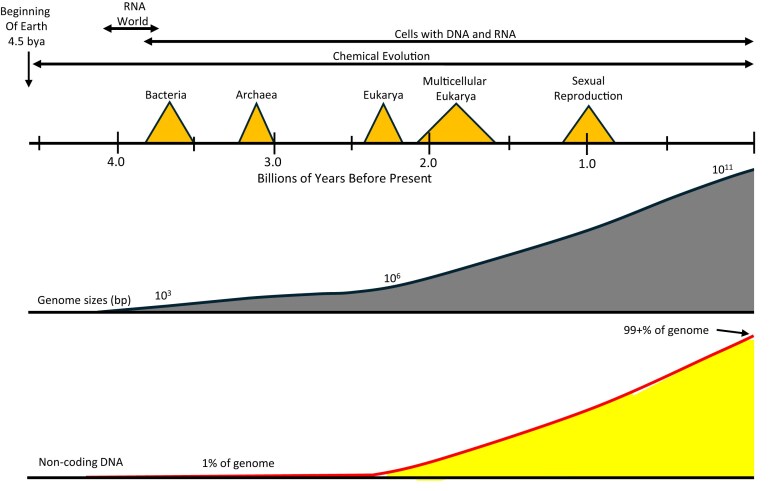
Increases of MGEs during genome evolution. The Earth coalesced just over 4.5 million years ago (bya). The first ∼500 million years on Earth were characterized by chemical evolution, primarily in pools and oceans that existed from ∼4.3 bya. Soon, some classes of molecules (primarily RNAs) were capable of self-replication and others displayed enzyme activity. This was the beginning of the RNA world. Some molecules formed membranes that surrounded and concentrated biochemicals, which became the first protocells, initiating biological evolution. Although DNA replaced RNA as the molecule of inheritance, RNAs played crucial roles in cells, as they do today. The first organisms were Bacteria (3.8 to 3.5 bya), followed by Archaea (3.2 to 3.0 bya). The orange triangles indicate the range of times when they first appeared. About 2.4 bya, a symbiotic event involving an archaeal cell and a bacterial cell occurred that led to the first eukaryotic cell. By 2.1 to 1.6 bya, multicellular eukaryotes were present, and by ∼1.0 bya sexual reproduction arose, separating germline from somatic cells. While bacterial and archaeal genomes remained relatively small (∼10^3^–10^7^ bp), eukaryotic genomes expanded greatly (∼10^6^–10^11^ bp), accompanied by tissue differentiation in plants, animals, some protists, and some red algae. Among eukaryotes, the ∼10^7^ range in genome size is at least 1000-fold greater than the range in gene number, with the genome comprising mainly noncoding DNA (lower graph). More details are provided in [5], Supplementary Figs S1 and S2, and Supplementary Table S1 [15].

Another type of MGE that moves without a dedicated transposase employs the well-studied process of genetic recombination. Recombination can lead to large changes in DNA sequence copy number, either in tightly clustered genomic locations or at widely dispersed spots. The result is massive increases (and decreases) in size of the nuclear genome (Fig. 1). The target site can be created either by site-specific enzymatic cuts or by nonspecific agents that cause DNA strand breaks. Such agents include radiation, oxidative, and glycation damage [14], and any process that requires strand cutting during repair of damage [15]. Damage repair may require error-prone DNA polymerases that may run for great distances, thereby creating sequence havoc that is unlikely to benefit the host organism. Such previously perplexing sequences, however, represent the proliferation of parasitic DNA.

The existence of a conflict between sequences that benefit the cell and sequences that benefit parasitic DNA clarifies several puzzling aspects of biology, as described below. Each aspect can now be seen as either consequences of DNA damage and repair or a means by which DNA damage is avoided.

## MGEs in prokaryotes and eukaryotes

The structural diversity of MGEs is much greater in bacteria than in eukaryotes, as are the types of transposon defense systems [3, 16]. In prokaryotes, the competition between parasite and host results in a genome containing some DNA representing the parasite, while most DNA exists as tightly packed genes that contribute to the fitness and survival of the host [2, 3]. In eukaryotes, the result is typically the reverse, with transposon DNA sequences accounting for most or nearly all of the genome [4]. The advent of distinct germline and somatic cell types in sexually reproducing eukaryotes may have created the means by which transposon DNA could occupy most of the genome (Fig. 1).

### Prokaryotes

Prokaryotic genomes have evolved for efficient perpetuation of the species, because genes benefiting the cell comprise most of the genome. Although bacterial viruses (phages) can sometimes carry genes from one host cell to another, phages are conventional viruses that parasitize and kill their host cells. For MGEs that are not viruses, however, an explanation for their existence was not obvious at the time of their discovery. Retrons are an example. They are genetic elements composed of noncoding RNA plus a specialized reverse transcriptase that typically generates a chimeric RNA–DNA molecule covalently joined by a 2′–5′-phosphodiester bond. Thirty-five years after their discovery, retrons were recognized as components of anti-phage defense systems that are widespread in bacteria [17]. Plasmids constitute another example. Although they can provide protection against antibiotics, and thus benefit the host cell under some conditions, it is now believed that plasmids are MGEs that exist for their own perpetuation [16]. Still other examples are the restriction–modification systems in bacteria. These exemplify the more general toxin–antitoxin systems that travel together from one host to another, multiplying and spreading within a population. Many diverse transposon defense systems, such as CRISPR [18, 19] and RNA editing [1, 20] (discussed below), are known in bacteria. What distinguishes bacterial defense systems from those in eukaryotes is the relatively small increment of genes, energy, and carbon allocation required to defend the cell against MGEs.

### Eukaryotes

The nucleus can contain large amounts of DNA that is probably not required (or even useful) for the organism. It is repetitive in the sense that two sequences are scored as similar to an extent that is defined by the investigator (Box 1). As detailed below, in some cases repetitive DNA is deleted during the early development of somatic cells from the zygote. In others, this large amount of DNA persists during development from zygote to adult somatic cell. However, its potential to scramble nuclear chromatin by recombination is avoided by introducing point mutations, chromatin compaction, or by causing mutations when “break-induced replication” (BIR) exposes single-stranded regions around the site of transposon integration [21]. Evidence that these mechanisms successfully maintain chromosome stability comes from cytological observation; chromosomes in normal (but not cancerous) cells can be identified across populations and generations. While the benefit to the organism of most nuclear DNA sequences is not known, chromosomes and genomes remain relatively intact, despite suffering continuous insertions by MGEs. This trade-off between genome stability and MGE instability has existed for billions of years (Fig. 1) and continues today.

Box 1. **Identifying repeated DNA sequences is imprecise**

Repeated DNA sequences may be either beneficial or detrimental to a cell. In either case, the cell must distinguish repetitive from nonrepetitive sequences, and the investigator attempts to do the same.

**Figure 2.**
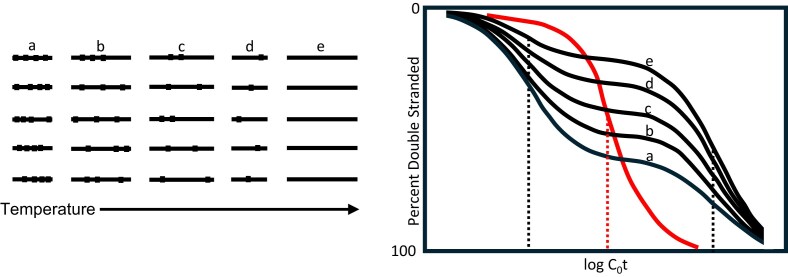
The left drawing shows five “families” (a–e) of double-stranded DNA (dsDNA) sequences unrelated to one another but related to those in its column. The marks represent sequence changes (mutations) among family members. DNA extracted from an organism is purified, fragmented to ∼400 bp, single strands are separated by heating, and the kinetics of strand reassociation monitored at different temperatures (right drawing). At low temperature, the sequence changes are insufficient to prevent a-family members from cross-hybridizing, so they reassociate rapidly: the “fast” component of the *C*_0_*t* curve (left part of the curves). When the reassociation temperature is raised, the sequence changes in the a-family are too great for the members to cross-react, and the a-family members now appear in the “slow” single-copy fraction of the genome. The reassociation rate (shown by the vertical dotted lines) for the fast component on the left is e.g. 10 000-fold greater than that for the slow component on the right, indicating a copy number of 10 000 for the fast component and one copy for the slow component. “*C*_0_*t*” means concentration of DNA at the start of reassociation multiplied by time of incubation and is shown on a log scale on the abscissa. The genome size of the eukaryote is calculated by dividing the *C*_0_*t* value for the slow component by that for the bacterial standard (vertical dotted line between those for the fast and slow components) multiplied by the genome size of the bacterial standard. The *C*_0_*t* value represents the probability for strands to reassociate.

Do our analytical methods correctly identify the sequences that are treated as repetitive within the cell? Repetitive DNA sequences were identified using DNA hybridization by analyzing the rate of reassociation of denatured DNA strands to form duplex molecules [22]. However, the degree of sequence similarity among sequences classified as “repeated” was shown to vary with the temperature used during reassociation, so that the fraction of a genome in the repetitive category varied with the stringency conditions chosen to perform the assay [23] (Fig. 2). This stringency uncertainty also applies to contemporary genome sequence analysis.

On the other hand, repeats too short for detection by nucleic acid hybridization *in vitro* do serve important functions in cells. In *Neurospora*, tandem arrays of dsDNA units containing repeats of 4–6 bp spaced at intervals of 11, 21, or 22 bp can pair to silence transposons in the processes of RIP (repeat-induced point mutation) and MSUD (meiotic silencing by unpaired DNA) [24, 25]. Furthermore, the codon–anticodon pairing of three nucleotides is the basis for specificity of the genetic code. In these examples, it is the nucleoprotein context that allows such short sequences to influence cellular metabolism.

The mitochondrial DNA (mtDNA) minicircles in trypanosomes contain sequences termed “genes” that are transcribed into guide RNAs for editing ribosomal RNAs (rRNAs) and messenger RNAs (mRNAs) for respiratory chain proteins. In *Trypanosoma brucei*, the number of such genes was estimated as 200–250 by DNA reassociation kinetics [26, 27] and ∼900–1200 by deep sequencing using a cutoff of 95% sequence identity [28], illustrating both the difficulties of gene identification and the extreme inefficiency of RNA editing to produce functional transcripts.

## MGEs are suppressed in prokaryotes but flourish in eukaryotes

A competition exists between DNA sequences that contribute to organism survival and those that contribute primarily to their own survival. Aspects of this competition are listed first, before they are addressed in detail.

- rRNA genes (rDNA) are the most frequently used genes in any organism; the rDNA is where evidence for the host/parasite conflict is strongest.
- Introns are found in nearly every gene in animals and plants; although they sometimes benefit the host cell, introns are MGEs that invaded genes for their own perpetuation.
- Nearly all repetitive DNA sequences in the nucleus, in both dispersed and tandem arrays (“satellite DNA”), derive from MGEs.
- The contribution of satellite and other repeated DNA sequences to the functions of chromosomal centromeres and telomeres represents a reliance on and an addiction to MGEs that ensure the perpetuation of these parasitic DNA elements.
- Since dispersed repetitive sequences could recombine to scramble chromosomal DNA molecules, such sequences must be identified and silenced for genome defense.
- RNA editing that creates transcripts that differ from their DNA templates arose as a transposon defense system.
- Genetic recombination is part of a DNA repair process that benefits the MGE more than the host organism.
- Sexual reproduction may have originated in eukaryotes as a way to spread MGEs.

## Ribosomal DNA contains transposon remnants

The rDNA units in a wide range of eukaryotes include regions that contain a diversity of introns, transposons, and DRs [29, 30]. The rDNA unit, which contains one of the most highly conserved sequences in eukaryotes, is adjacent to one of the most highly variable sequences, the intergenic spacer (IGS). In most eukaryotes, this unit is repeated in tandem many times to provide hundreds of thousands to millions of ribosomes. Until recently, the heterogeneity of IGS sequences was so variable and repetitive that it was largely ignored in genome annotations, appearing as blank segments in maps. However, we now know that IGSs are filled with two sequence types derived from retrotransposons: (i) truncated retrotransposons flanked by short DRs and (ii) far more numerous short DRs, as well as tandem DRs called microsatellites comprising different sequences evidently formed after the intervening retrotransposon element was excised. This process that would bring the flanking DRs together in tandem [29] is an idea developed for mammalian retroviruses by Deininger [31]. The fact that IGSs are filled with transposon remnants indicates that IGSs have been sites of frequent MGE insertion; the many rDNA copies have been occupied by very many MGEs.

Not only have MGEs “colonized” the IGS portion of the rDNA, but they have even inserted within the rRNA genes themselves. In a variety of eukaryotes, the rRNA-coding segments contain transposon inserts that prevent either the large or the small rRNA molecule from functioning in protein synthesis [32]. Some of these inserts may be spliced out of the rRNAs resulting in functional rRNAs [30]. However, the insert-containing rDNA units often outnumber the units without an insert, so that most rDNA cannot produce functional ribosomes. Another curiosity is that the copy number of rDNA per genome can vary greatly among individuals in a population: 7-fold for some salamanders, 10-fold for some newts, as much as 95-fold for the plant *Vicia faba*, and even 12-fold among tissues in a single individual of *V. faba* [33]. Cells have adjusted to frequent MGE insertions by maintaining excess rDNA copies and by removing defective copies through concerted evolution to retain sufficient functional copies to produce the required number of ribosomes [34]. Most remarkable are the electron microscopic images (Miller spreads) from the green alga *Batophora oerstedii* [35, 36]. Material from an isolated nucleolus shows tandemly repeating rDNA units comprising coding segments spaced by an IGS, as is typical of many eukaryotes, whereas other tandemly repeating rDNA units in the *same nucleolus* have no visible IGS. Other rDNA unit arrangements are not uncommon, including head-to-head, tail-to-tail, truncated, and extended transcription segments. The presence, absence, or orientation of an IGS does not affect the transcription and production of rRNA. In summary, these curious data are not consistent with the idea that the number and transcription of rDNA units contribute only to organismal survival. The data are, however, consistent with the proliferation of the MGEs themselves, especially retrotransposons that proliferate via an RNA intermediate.

In plants, animals, fungi, and protists, the IGS segments contain repetitive elements of various lengths, including DRs that range from a few to several hundred base pairs. The number of the longer DRs within IGSs can vary greatly, even in a single nucleolus. In wild-type individuals, the properties of repeats in the IGS, as well as the copy number and arrangements of rDNA units, appear to represent genetic polymorphisms. Thus, as with the algae described earlier, for other eukaryotes the lengths of the IGS regions are not important for the production of rRNAs in the organism [35, 36]. In prokaryotes, there is no IGS, and each of several rRNA gene sets is transcribed in proportion to the need for ribosomes as growth conditions change.

The data above indicate that rDNA intergenic sequences may provide little or no benefit to the organism and that the copy number of non-defective rDNA units, above some minimal level, may be a wasteful expenditure of energy. One explanation is that parasitic DNA sequences of several kinds are the main beneficiary and that events attending the insertion and excision of DNA segments allow MGEs to expand in eukaryotes, but they are highly constrained in prokaryotes.

## Introns are MGEs

In a study of 213 introns (group I, group II, group III, spliceosomal, archaeal, and twintrons) from a broad range of organisms, we found DRs in the vicinity of the intron/exon borders [12]. Flanking DRs were found in all 213 introns: 44% were at or within 1 bp of an intron/exon border and >80% were within 10 bp of a border. When the positions of the borders were compared to the positions of the DRs for each of the 213 introns, a minority were found where the DRs and the pair of borders corresponded precisely. We concluded that for most of the 213 introns, the RNA splice products would differ from the RNA encoded by the gene before the intron inserted into that gene. Another conclusion from this study was that because DRs are characteristic of MGE insertion, and some transposons share features with group I or group II introns, introns and transposons are members of a group of parasitic MGEs. In dinoflagellate genomes, “introners” were identified as spliceosomal introns mobilized by specialized transposable elements [37], again indicating a strong connection between introns and transposons.

R1 and R2 are retrotransposons (related types of MGEs) located in some, but not all, small subunit and large subunit rRNA gene repeats in many animals. While concerted evolution, a process that essentially homogenizes rDNA repeats [34], often removes some of them, they rapidly reappear [38–40]. When rDNA copy numbers are large, these retrotransposons are inactive. When copy numbers are small, repression of R1 and R2 transcription is lifted, and R1 and R2 actively insert into additional rDNA repeats, thereby assuring their propagation. Entry into each new rDNA site, however, involves a double-strand break that leads to additional damage to the surrounding sequences. Whereas one result of these insertions is an increase in number of rDNA units that might benefit the host cell, another is an increase in number of the parasitic R1 and R2 elements.

The coding regions of the rDNA in a wide range of eukaryotes have experienced numerous MGE (i.e. intron) insertions during the past 3.5 billion years [30]. After analyzing the numbers and positions of introns within the small and large rRNA genes of eukaryotes, it was proposed that contemporary rRNA genes were created by stepwise assembly of preexisting sequence modules [41, 42] caused by MGE insertions and ligations [30]. We have analyzed the sequences flanking the proposed most recent rRNA gene modules to test the hypothesis that these modules were added by intron insertion. DRs were present in 17 of the 17 segments examined (Supplementary Figs. S3 and S4), as well as in the one transfer RNA (tRNA) model that was examined (Supplementary Fig. S5). Thus, the segments of rRNA and tRNA genes appear to have been added via introns (i.e. MGEs), supporting our hypothesis. Since introns are also sites for additional intron insertions [43], these additions to rRNA and tRNA genes are probably frequent; they either cause deleterious effects or rarely beneficial effects by adding expansion segments to the ribosome. In sum, rDNA loci have experienced frequent insertions by MGEs that may be continuing. While a host gains occasional benefit, the ultimate beneficiaries are the MGEs. In fact, most eukaryotic genomes may now consist largely of MGEs and their remnants (Fig. 1 and Supplementary Table S1).

The difficulty in identifying an MGE in a sequenced genome, as exemplified by retrons in prokaryotes, also applies to eukaryotes. A transposon requires a transposase (or similar activity) for mobility or the ability to locate breaks in DNA in which to integrate. It took more than a decade of research to discover that the simple-sequence MITE transposons were nonautonomous elements that utilize transposases encoded by autonomous transposons not classified as MITEs [13]. Similarly, it took years before the germline-limited “internal eliminated sequences” in ciliates were recognized as decayed transposon sequences [32, 44]. Also, until recently, the vast majority of introns (devoid of transposase genes) were not recognized as MGEs [12].

## Clustered and dispersed repetitive DNA sequences in the nucleus represent MGEs

### Chromatin diminution in eukaryotes

One way to allow sequences favoring survival of the organism to coexist with sequences favoring themselves is to segregate the competitors to different compartments. The earliest description of the elimination of vast amounts of DNA during the transition from germline cells to somatic cells came from cytological observations with the parasitic nematode *Parascaris univalens* [44, 45]. Telomeric regions of the chromosomes (seen as darkly staining regions termed heterochromatin) were removed leading to chromosomes of smaller size in somatic cells. More recently, it was shown that this “programmed DNA elimination” removed up to 90% of the germline DNA in *Parascaris* and two related genera, most of which was in the form of tandem repeats (satellite DNA, satDNA) having sequences that differed in repeat length and sequence among the genera, as well as some 1000–2000 genes [46, 47]. While DNA elimination may be a mechanism for preventing the expression of these genes in somatic cells, the data are also consistent with removal of a potential threat to chromosome integrity posed by transposon DNA.

**Figure 3.**
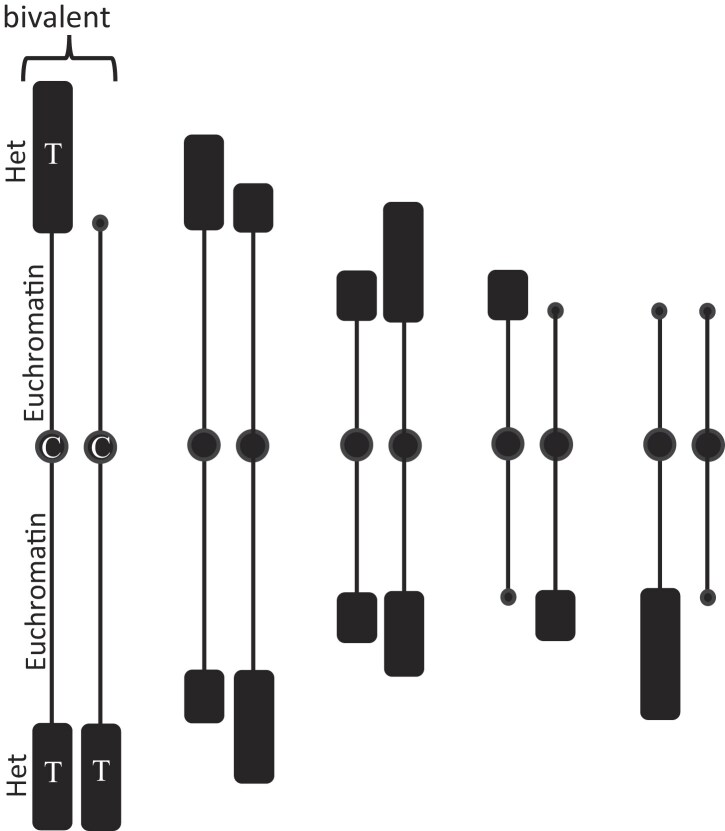
Chromosome diminution. Diagrams of bivalent chromosomes from *C. furcifer* showing varying patterns of chromosome diminution during meiosis. Note that the heterochromatin (Het) is preferentially removed, while leaving the euchromatin intact. C = centromere; T = telomere. (Examples from [48]).

In a cytological *tour de force*, Beermann [48] reported the elimination of Het from all germline chromosomes (2*n* = 22) during the fourth, fifth, and sixth cleavage divisions following fertilization for three species of *Cyclops* (copepods). The Feulgen-staining blobs of Het were removed from the telomeres (*Cyclops divulsus*), centromeres and telomeres (*Cyclops furcifer*, Fig. 3), and numerous small regions along the chromosomes (*Cyclops strenuus*), without affecting the continuity of the remaining euchromatin. The process was thought to be similar to the excision of the lambda prophage from the *Escherichia coli* chromosome during the lytic phase of phage infection. For *C. furcifer*, electron microscopy of early-diminution nuclei showed closed rings of chromatin of various sizes [49], consistent with the lambda analogy. Various numbers of satellite-type DNA units per excision event were observed. Three decades later, ∼94% of the germline genome that was eliminated in a fourth species (*C. kolensis*) was found as circular molecules of Het containing repetitive motifs of 15–300 bp, some as microsatellites, some as retrotransposons, and some as rRNA gene clusters [50]. We posit that this pattern of retention/diminution illustrates the competition between parasitic and host-beneficial sequences. Retention in the metabolically quiescent germline ensures inheritance of the MGEs, while diminution provides two benefits to the metabolically active somatic cells: reducing the threat to scramble somatic cell chromosomes by recombination among repeated sequences and avoiding the cost of replicating the eliminated MGE. Prior to diminution, the amount of heterochromatin in meiotically paired chromosomes (bivalents) was extensively polymorphic for individual animals in wild populations of *C. furcifer* and *C. strenuous*. This polymorphism may indicate that some members of these populations have avoided the insertion of MGEs, whereas other members have not.

Ciliates are unicells with a macronucleus required for day-to-day life (somatic activities) and a micronucleus comprising the germline, which is used in sexual conjugation [32]. The micronucleus is mostly transcriptionally silent until sexual conjugation, and its transposon-like DNA, minisatellites, and other repetitive DNA sequences are eliminated when the macronucleus develops from the micronucleus. The complex genomic rearrangements are guided by small RNA molecules, with the end result being the same as in the previous examples: potentially disastrous recombination among numerous repetitive DNA sequences is avoided. Despite seemingly costly nucleic acid gymnastics, both the host cell and the MGEs are maintained because they depend on each other. The host requires the small RNAs transcribed from the MGE to avoid transferring repetitive sequences to the macronucleus, and the MGE requires the host for its perpetuation. Such mutual dependency in ciliates is similar to that in prokaryotes, where it is known as a toxin–antitoxin transposon defense system [16].

In each of these examples (nematodes, copepods, and ciliates), the somatic cells divide many times during the life cycle after removal of the germline-limited chromatin that contains highly repetitive satDNA. Thus, it is apparent that these repetitive sequences, which are present in much higher copy number than any genic sequences that may also be eliminated, are not needed for chromosomal function or cell division in somatic cells. Furthermore, satDNA is difficult to replicate accurately (see below). It follows that for each example, there is a strong adaptive reason to prevent satDNA from reaching the soma. The likely reason is that these satDNAs are a simple form or product of MGEs that pose a threat to somatic cells and that chromatin diminution removes that threat. We now turn toward other ways to avoid the threat of repetitive DNA.

### Repetitive DNA in clustered and dispersed arrays represent parasitic sequences

SatDNA is found in prokaryotes and eukaryotes. A tandem arrangement of repeating units—usually imperfectly repeated units—is the best descriptor of satDNA [29]. In bacteria, the repeat unit is typically short (<10 bp [51]), whereas in eukaryotes it can be short or long (several kb in nuclear rDNA). SatDNA can comprise most of a chromosome or genome. The fraction of a bacterial genome comprising satDNA is too small to noticeably alter the single-component appearance in a *C*_0_*t* curve (Box 1). A long tandem array of units creates an excellent site for recombination that would destabilize the genome if that recombination were not restrained. Furthermore, a tandem array of short units is difficult to replicate accurately, leading to DNA polymerase stalling and slippage, template strand switching, and DNA strand breaks [20] that create insertion opportunities for introns and other MGEs that may be detrimental to the host cell.

Britten and Kohne [22] used DNA hybridization kinetics to identify repeated DNA sequences. These were similar, but not identical sequences considered at the time to be important for development in animals and plants [52]. However, the criteria used to classify sequences as repetitive depended on stringency conditions chosen to perform the assay, so that an investigator might mistakenly assign repeat-related properties to sequences treated as unrelated by the cell (Box 1). The concept of selfish DNA [53, 54] provided an alternative reason for the existence of many “copies” of a DNA sequence; subsequent analysis revealed that most of the repeated DNA originated as MGEs [4] that decrease fitness of an organism [55, 56]. A particularly instructive example is a single family of repetitive sequences of the non-LTR type of retrotransposon present in ∼240 000 copies in the genome of *Lilium speciosum* that is undetectable in *Lilium longiflorum* [57]. It is difficult to imagine how these nine *Arabidopsis*-genome equivalents of DNA contribute to the well-being of one lily but not the other. This large amount of additional DNA may reflect susceptibility to insertion by this genomic parasite in *L. speciosum*, whereas *L. longiflorum* is resistant to this parasite. If so, then the biochemistry of genome defense distinguishes one lily from the other, rather than novel gene regulation orchestrated by repeated DNA. In conclusion, clustered and dispersed repetitive DNA sequences originating from genomic parasites represent a potential threat to chromosome stability.

### Transposon defense systems

How does the host cell defend against repetitive DNA persistently scattered throughout the nuclear chromosomes in most eukaryotes? Several mechanisms are used by the filamentous fungus *Neurospora crassa* that cause either genetic change by mutagenesis (RIP in *Neurospora* and related fungi) or epigenetic change that silences DNA by chromatin compaction (Box 2). The repressed chromatin can, however, spread beyond the transposon and is usually detrimental for the host cell [58]. Genetic mechanisms are precise and permanent, although they are relatively slow to respond to environmental change. Epigenetic mechanisms include RNA editing (described below) and RNA interference (RNAi), and although these are imprecise and temporary, epigenetic mechanisms can flexibly alter gene expression during development of plants and animals. “The proclivity of RNAi to recognize transposon sequences, which is the proposed ancestral function of the pathway, can of course serve to protect the integrity of the genome from these potential invaders, but this function has clearly been adapted for other purposes” [59]. A wide variety of genetic and epigenetic processes appear to have originated as mechanisms to control the spread of MGEs.

Box 2. **The diversity of MGE defense systems in eukaryotes**

Transposon silencing may be accomplished in several ways, as seen in *N. crassa*. RIP and MSUD are the best-understood examples, whereas the way in which RNA editing contributes to transposon defense was unclear [56, 60, 61]. In RIP and MSUD, the site to be targeted for silencing is identified by a process in which chromosomal DNA pairing in meiosis is achieved by pairing of homologous dsDNA segments (in C-form DNA [62]), rather than the well-studied recombination process involving single-stranded DNA and a RecA-type of protein [63]. RIP occurs prior to meiosis and triggers C-to-T mutations in both clustered and dispersed transposons within the nucleus; MSUD occurs later in development, during the onset of meiosis [64], and operates on short homologous sequences spaced widely by non-homologous sequences. In contrast to RIP, MSUD utilizes small RNA molecules and is similar to the epigenetic process of post-transcriptional gene silencing (PTGS) found in yeasts, animals, and plants [58, 59]. The distinction between silencing at the genetic level (RIP) and the epigenetic level (MSUD and PTGS) is important because epigenetic marks (e.g. methylation on DNA and histones) are typically erased and reestablished each generation, whereas mutations are essentially permanent. RNA editing operates on transcripts from already-mutated DNA and is also permanent, despite the heavy price the transposon exacts from the host organism to maintain and express many additional genes.

How did editing arise and why haven’t PTGS mechanisms replaced editing? Editing as a defense system against MGEs originated in prokaryotes (Bacteria and Archaea), was inherited by eukaryotes during endosymbiosis, and then diversified to the forms we now find scattered among eukaryotic lineages according to the following scenario. Sexual reproduction, which is absent in prokaryotes but present in almost all eukaryotes, created a dichotomy between germline and somatic cells such that a proposed cost saving in DNA repair could be realized [65]. Sex also opened a new route for the spread of transposons through a population that required novel defense responses from the host (including chromatin diminution, RIP, and MSUD/PTGS) that allow the host to retain control of its heredity, as well as RNA editing where the transposon controls heredity. RNA editing in *Neurospora* was deemed “functionally related to RIP and MSUD” without clarifying this relationship, whereas expanding protein diversity during sexual reproduction was considered the main advantage of RNA editing [61, 66]. Emphasis on such derived beneficial effects of editing may have obscured its origin as an instrument for transposon defense, which is not mentioned in recent reviews of editing [60, 67–72].

Epigenetic mechanisms alone are sufficient for silencing nuclear genes in yeasts, animals, and plants [59, 60] and thus may be considered as having largely replaced RNA editing in these lineages. In *Arabidopsis*, however, whereas silencing of nuclear genes is accomplished by RNA-guided DNA methylation [73], PPR (pentatricopeptide repeat) protein-guided editing operates in the mitochondria and plastids. In the mitochondria of trypanosomatids editing is RNA-guided, whereas PPR proteins are used to stabilize transcripts for translation but not to guide editing [67]. The reasons for these differences are not presently known.

When a pathogenic organism or virus spreads through a population, it may kill most individuals, although a few survive infection. Similarly, an MGE may kill most cells, but survivors may have silenced the MGE. Later, the formerly deleterious MGE or its derivative may be repurposed for the benefit of the host, a process known as exaptation (also termed “domestication” [74]). An example is the use of a retrotransposon sequence to provide an essential function during early mammalian embryo development [75]. Thus, an epigenetic defense mechanism may evolve to become a genetic mechanism. In either case, the MGE proliferates, as does the surviving host.

The inefficiency of RNA editing (hundreds to thousands of genes required to produce correct ribonucleotide sequences among many incorrectly edited transcripts) may be why RNA editing was not previously recognized as a defense mechanism against MGEs. The unquestioned benefits of RNA editing in the nucleus have apparently obscured the original purpose of RNA editing in bacteria as defense against DNA damage attending MGE insertion. That purpose is most clearly evident in contemporary genomes of mitochondria and plastids.

Mitochondria and plastids have also accumulated MGEs, and their genomes reveal this in many lineages. There are four properties of organellar DNA (orgDNA) that are unusual in biology:

- 1. In some organisms, including most animals, the organellar chromosomes comprise tightly packed genes without intergenic spacers, as found in their bacterial progenitors. In others, including plants and fungi, genes comprise a small fraction of the chromosomes, and intergenic spacers comprising transposons and introns account for most sequences in chromosomal DNA molecules.
- 2. Unlike the DNA in the nucleus and bacteria, orgDNA is unstable. In some organisms, the orgDNA turns over within persistent organelles (e.g. the green alga *Chlamydomonas*), whereas in others, the entire organelle is degraded (mammals) or removed from the cell (some ciliates and algae). In maize, orgDNA molecules in germline cells of the basal meristem of the shoot are long, whereas orgDNA molecules degrade rapidly in leaf cells that develop from the basal meristem.
- 3. Sequence analysis reveals small orgDNA molecules in circular or hairpin forms carrying one gene, part of one gene, or no genes (“empty chromosomes”). These small molecules may comprise the entire organellar genome or exist in higher copy number than longer molecules representing a conventional genome.
- 4. Heredity of mitochondrial and plastid traits differs from Mendelian inheritance in the nucleus. The difference results from high levels of DNA damage from both oxidative and glycation reactions and low levels of damage defense, including inefficient RNA editing (Box 3).

Box 3. **Chromosomal DNA molecules in mitochondria and plastids: form, size, stability, and dependence on RNA editing**

The history of research on mtDNA and plastid DNA (ptDNA) is instructive [76–79]. Early on, these chromosomal DNA molecules were generally thought to be circular and the size of the unit genome. This conclusion was based on two observations: mtDNA molecules in animals from several phyla are invariably circles of ∼16 kb when examined by electron microscopy; and *E. coli* chromosomal DNA was thought to be circular and representative of the bacterial endosymbiont that evolved into the mitochondrion. Later, chromosomal DNA from chloroplasts and mitochondria of flowering plants (angiosperms) was also reported to be circular, though the size of the mtDNA circles differed greatly from the size of the genome determined subsequently. Whereas the ptDNA circles were of genome size, the circles comprised only a small fraction of all ptDNA molecules (most of which were excluded before analysis), and the ptDNA molecules in germline cells were larger than the size of the genome and found in variable sizes and branching forms. When algae and additional land plants were studied, the genic content of organellar genomes remained relatively constant among species, but their genome sizes could vary as much as 5-fold for plastids and 30-fold for mitochondria due mainly to transposons, introns, and unclassified noncoding sequences [80–83].

In brief, it took several decades to identify the mitochondrial and plastid chromosomal DNA molecules that are inherited during phylogeny. Reasons for this delay include inadequate taxon and tissue sampling, faulty assumptions concerning circular or linear forms, reliance on mapping and sequencing data rather than physical analysis of DNA molecules, and failure to appreciate the potential of sequences to behave parasitically.

Chromosomal DNA molecules are stably maintained throughout development for animals and plants, as well as bacteria growing on rich or minimal media, indicating that DNA repair operates well. This is not true for orgDNA. The best example comes from the single-celled alga *Euglena gracilis*, where ptDNA and mtDNA are unstable (half-lives of 1.6 and 1.7 cell doublings, respectively), whereas nuclear DNA turnover could not be detected [84, 85]. Cellular and organellar development in maize proceeds from the shoot basal meristem (analogous to embryonic stem cells in animals) containing promitochondria and proplastids to the leaf blade containing mitochondria and chloroplasts. During this development, orgDNA molecules change from long, undegraded to highly fragmented molecules, oxidative and glycation damage increases, and damage defenses decrease. The damaged orgDNA molecules are not replaced, but are “abandoned” [14], as are entire organelles eliminated from motile sperm in plants and algae, leading to uniparental inheritance [86].

Three lessons can be drawn from orgDNA research. First, taxon sampling errors can lead to erroneous generalities that persist for decades. Second, the large variation in genome size—even among closely related organisms and attributable to MGEs—applies not only to the nucleus but also to mitochondria and plastids. Third, whereas the chromosomal DNA molecules in the diploid nucleus are constant in form and size during development—probably due to checkpoint control of cell division [87]—orgDNA molecules can be highly damaged and degraded in robust somatic cells. We now move to the possible relationship between RNA editing and transposon defense.

The sporadic appearance of editing during the phylogeny of eukaryotes [69] is consistent with editing as a transposon defense system. When a mutational event alters the standard coding version of a gene, metabolism operates inefficiently until the individual either dies or is saved by a rectifying event, such as editing. At this point, there is no turning back regardless of imprecision, additional energy (see main text), and additional required genes—thousands for some land plants [70]. The change is genetic, not epigenetic, and the condition laid down by the MGE is non-negotiable: *Allow me to take over your genome or perish*. Returning to the original pre-editing DNA sequence now requires a back-mutation. The sporadic occurrence of RNA editing during phylogeny may thus be understood as individual results of an “arms race” between invading MGE and host resisting the invasion, as extensively studied in prokaryotes [3, 16]. RNA editing can be found in one lineage but not in a neighboring lineage: in land plants but not green algae [69]; for mitochondria and plastids of most land plants but not the liverwort *Marchantia* [70]; and to greatly different degrees for the mitochondria among three subspecies of *Trypanosoma brucei*: *brucei, equiperdum*, and *evansi* [88]. Such editing variation among organisms may reflect the variation in the ability to avoid infection by conventional viruses and transposons (e.g. *L. speciosum* and *L. longiflorum*), according to the proposal that editing is one way to defend against MGEs. The biochemical details of such defense may not be known, but at least for conventional viruses, sporadic resistance to infection is generally accepted without surprise. The outer covering of viruses allows them to move via an extracellular route, whereas transposons can spread only by cell-to-cell contact [89]. Perhaps sexual reproduction originated as a means by which parasitic transposons could greatly expand their host range via the fusion of gametes.

## Types and consequences of RNA editing

In what may be one of the most remarkable observations in genetics research, Benne *et al.* [90] reported that the nucleotide sequences in mtDNA of trypanosomes do not contain the coding information for the amino acid sequence of some mitochondrial proteins unless the transcript sequence is changed. The sequence-altering process, known as RNA editing, was subsequently found for genes in the nucleus, mitochondria, and plastids for some species, but not for other related species [69]. We postulate that RNA editing is a transposon defense mechanism, as described below.

There are several ways in which RNAs are known to be edited. In trypanosomes, editing involves the deletion and addition of uridine (U) residues from the mtDNA transcripts. The classic example of editing in humans is the deamination of cytidine to produce uridine (C-to-U) in mRNA from the nuclear gene encoding apolipoprotein B, which is important in adaptive immunity. The key enzyme is in the APOBEC family of proteins, although members of this large family of proteins also serve to defend the cell against MGEs, illustrating the dual beneficial functions of protection against genomic parasites and control of cellular gene expression [91]. In another type of editing, adenosine deaminase (ADAR) converts adenosine to inosine (A-to-I) to both protect against parasites and influence gene expression [92]. A-to-I editing of nuclear gene transcripts also benefits filamentous fungi that employ an ADAR-like protein [93]. In plant cytoplasmic organelles, C-to-U deamination is the major editing event, and PPR proteins are used to target the editing sites required for proper respiration and photosynthesis [70].

The numerous hypotheses for RNA editing fall mainly into two groups. The first posits benefit to the organism, such as added flexibility in gene expression during environmental and developmental change or defense against DNA damage and mutations. The second is a concept called “constructive neutral evolution” (CNE): editing originated without benefit to the organism. Gray [94] asked whether a *general principle* exists that could explain the emergence of editing and its diverse RNA-modifying mechanisms in different organisms. We now review data that may have revealed this principle.

Irrespective of the type of nucleotide alteration or the proteins performing editing, there are clear examples of how editing is helpful or essential to organism viability; these weigh against the concept of CNE. However, it is the origin of editing, rather than its subsequent evolutionary adaptations that must first be addressed. Under CNE, only components of editing systems, such as adenosine and cytidine deaminases, rather than fully functional editing systems were present in the prokaryotic ancestors of eukaryotes. However, editing has been identified in bacteria, where it is part of a toxin–antitoxin system [20, 95]. A two-gene cassette consisting of a divergent adenosine deaminase and an ATPase was found clustered in genomic defense “islands” with other antiviral systems, including restriction–modification and CRISPR–Cas [1]. RNA editing occurred only when both the phage and the defense system were present, indicating that the editing-dependent defense mechanism was activated by phage infection. Editing also increases transcript diversity and flexibility and expands the bacterial proteome. The same secondary beneficial functions are more extensively documented in eukaryotes [20, 96, 97]. In conclusion, RNA editing as an MGE-defense mechanism was likely present in the first eukaryotes, and this defense is still its primary function. However, post-transcriptional gene silencing has augmented or even replaced editing for transposon defense in some eukaryotes.

In the CNE concept, components of RNA editing were present before they were recruited for editing [94], but the data reviewed here are inconsistent with this idea. Editing is fully functional and adaptive in prokaryotes, the ancestors of eukaryotes. Furthermore, there is nothing “neutral” about retrons, chromatin diminution, or RNA editing. These are instruments of defense against MGEs, and that may be the elusive *general principle* of RNA editing.

To summarize, the nuclear genome is composed mainly of parasitic DNA in both clustered and dispersed arrays. After systems evolved to prevent parasitic DNA from killing the host organism, parasitic DNA had little effect on organismal phenotype. In eukaryotes, RNA editing has not generally been classified as a transposon defense system for two reasons. First, there are clear benefits of editing, a prime example of which is the role of editing in the evolution of the vertebrate immune system [91]. However, it was proposed that the original transposon defense function of editing was modified to direct DNA repair leading to somatic hypermutation and antibody diversity [98]. The second reason is the requirement of many additional nuclear genes to produce the same transcripts encoded in the DNA of related organisms that do not require RNA editing.

## The cost of maintaining DNA representing MGEs

The entry of an MGE at a DNA insertion site can be initiated in three ways, each creating damage that must later be repaired: (i) an endonuclease cuts the insertion site; (ii) by non-sequence-specific agents such as radiation and mutagens; and (iii) when DNA replication stalls at hard-to-replicate satDNA sequences [32] (described earlier). Given the abundance of satellite sequences in eukaryotes, possibility (iii) may represent the major genome-destabilizing effect of MGEs in eukaryotes.

In order for RNA editing to compensate for a DNA sequence change (a mutation), the correct site within the transcript must be identified. We currently know of two mechanisms for identifying that site; both are extremely inefficient. Most of the biochemical information for small-RNA-guided editing comes from the study of the mitochondria of trypanosomatids [99]. In land plants, however, editing-site selection in mitochondria and plastids is accomplished by nucleus-encoded PPR proteins rather than small RNAs, and the number of different PPRs employed can exceed a thousand per cell [70]. For both RNA-guided and protein-guided editing, there are many more improperly edited than properly edited transcripts [67, 70]. The genetic “cost” of maintaining these editing-related genes and gene products must be enormous when compared to the original pre-editing DNA sequence (Box 3). Furthermore, that cost varies greatly between organismal lineages that did or did not carry the mutation at the pre-editing sequence. One additional cost is the ceding of control of heredity to the parasitic MGE (Box 4). For these costs to be borne by the host suggests that MGEs have prevailed in the competition for occupancy of the eukaryotic genome.

Box 4. **RNA editing and the relationship between mobile genetic element and host**

The mechanisms for identifying transposon sequences involved in gene silencing have been elucidated largely in organisms with well-developed procedures for genetic analysis and relatively small genomes, with *Neurospora* as the prime example. In *N. crassa*, 9000 protein-coding genes involved in diverse processes had at least one recoding editing site [61].

One kind of transposon defense may be important in organisms with large genomes (e.g. salamanders and lilies). Consider the insertion of a linear DNA retrotransposon into one of the thousands of target sites in the genome of *L. speciosum* (see main text). BIR, also known as recombination-dependent replication, is a nonstandard replication process initiated by one broken end of a DNA molecule invading a homologous template, followed by migrating bubble replication and long stretches of single-stranded DNA leading to frequent mutations [100–102]. Whereas RIP and MSUD protect the genome in a recombination-*independent* fashion, a BIR-like mechanism might operate on a wide scale in large genomes, with two interesting consequences. The new DNA sequence changes in intergenic regions (derived mainly from MGEs that account for most of the chromosome) would protect against chromosome scrambling by recombination, as does RIP. If the effect of the transposon insertion “spreads” to a neighboring off-target essential gene, RNA editing could be used to correct the defective transcript in somatic cells and rescue the host. Editing could also expand the proteome epigenetically in somatic cells without ceding further control of heredity to the MGE in the germline cells.

## The benefits of recombination and sex

There are pros and cons of meiotic sex. Some authors conclude that the overwhelming prevalence of sexual reproduction in eukaryotes is strong evidence that sex is beneficial. Others disagree and feel that the various drawbacks of sex require some other explanation [103]. Here, we offer a possible explanation for sex that involves the parasitic properties of MGEs. In prokaryotes, where sex is absent, MGEs are almost completely excluded from the chromosome. Sex associated with early eukaryotes allowed MGEs to spread throughout the population. After a germline/soma dichotomy arose (Fig. 1), MGEs gained a second advantage by using the low-metabolism, low-ROS germline to allow them to proliferate and spread [65]. MGEs may have been the initial beneficiaries of sex. Later, the benefit of chromosome and exon shuffling allowed complex eukaryotes to flourish and their nuclear genomes, transcriptomes, and proteomes to expand greatly.

In order to understand our possible explanation for sex, we need to consider the following. *RecA* was the first recombination gene discovered in *E. coli*, where repair of DNA damage was taken as its function [104]. In eukaryotes, that gene is called *RAD51* and repair is still its main role in maintaining cell viability, although population diversity generated by sexual reproduction may also benefit the species. *RAD51*-related genes are also used in additional functions, including pairing of homologous chromosomes at meiosis I during sexual reproduction. Pairing is typically accomplished by deliberate dsDNA cutting—an act of damage that must later be repaired—so that crossovers may properly align homologous chromosomes for successful meiosis. Oddly, only a few percent of the dsDNA breaks lead to crossovers, with most dsDNA breaks not contributing to chromosome alignment but still requiring expensive repair [105, 106]. This biochemical inefficiency is one reason why the benefits of sex (with separate germline and somatic cells) have been debated for decades. Another reason concerns how “anticipated” benefits for future generations can affect survival of the individual attempting to reproduce now. These and other advantages/disadvantages of sex have been reviewed [103].

We note two additional points of relevance. First, a RecA family member involved in meiosis pairing in yeast and human is DMC1, which can tolerate mismatches during heteroduplex formation [107], rather than the less-mismatch-tolerant RAD51 used to repair DNA damage in nonmeiotic cells. The use of this seemingly “second-class” recombinase for crossing over at meiosis is one unexplained aspect of sex. The second puzzling aspect of sex is found among species of *Drosophila*. *Drosophila melanogaster* females employ crossovers at meiosis to align homologous chromosomes before gametes are produced. In male *D. melanogaster*, however, no crossing over at meiosis was found and yet sperm are produced [108]. Singh [109] concluded that recombination “occurs at a very low rate” in *D*. *melanogaster* and five other *Drosophila* species. However, in a seventh species (*Drosophila ananassae*), the recombination rate was high and depended on numerous factors, including the strain of this species and even when brothers were tested. These data for DMC1 and for *Drosophila* challenge the notion that at its inception the benefits of sex outweighed the drawbacks of sex. However, the data are consistent with the notion that the initial benefit of sex was to spread parasitic MGEs through a population.

## Concluding remarks

From principles of population genetics, natural selection is strongest when effective population size is large, as commonly found in rapidly dividing prokaryotes and some rapidly dividing eukaryotes (e.g. yeast). When populations are small, as found in large animals and plants with long generation times, mildly deleterious traits may escape selection, allowing parasitic DNA sequences to accumulate in some genomes to astounding levels. Another factor affecting genome occupancy—and the one on which we have focused—is the competition between DNA sequences that benefit the host organism (e.g. genes) and parasitic sequences that primarily benefit themselves (MGEs, including conventional viruses, transposons, introns, and satellite DNAs). We posit that this competition best explains the large quantitative differences among species, individuals, and tissues within an individual described above (e.g. rDNA differences in *Vicia faba*, RNA editing differences among *Trypanosoma brucei* subspecies, and chromatin diminution in *Cyclops* in Fig. 3). Genomic parasites may occasionally persist and even become “domesticated” to benefit the host. Such instances are probably rare; we know of them because of their positive or negative phenotypic effects.

The existence of conventional pathogens (cells and viruses) is not surprising; they persist because they successfully parasitize their hosts. Transposons and introns are MGEs that persist for the same reason, but they are confined within cells. While prokaryotes largely suppress MGEs, suppression is weak in eukaryotes. Even when defenses succeed in maintaining host viability, MGE remnants remain and may eventually occupy most of the host genome. Although host genomes may occasionally benefit from MGE insertion (e.g. alternative splicing), the fact that eukaryotic genomes are replete with MGEs and their remnants indicates that MGEs are the primary beneficiaries of the host–parasite relationship.

Two ways exist by which eukaryotes defend the genome against invading MGEs. One is to employ RNAi to suppress MGEs. This epigenetic way is imprecise and may spread to off-target sequences. It is also only temporary because the repressive marks, such as methylation and acetylation, are removed each generation. The second way, chromatin diminution, is genetic. It precisely removes repeated sequences from somatic cell derivatives; and it persists through many somatic cell generations.

Meiotic sex involving recombination is found in many, but not all eukaryotes. Recombination requires breaking DNA strands, damage that must be repaired. One benefit of recombination is that it provides organisms with greater variability upon which survival may depend. Another benefit accrues to MGEs because they insert at sites where DNA strands are broken. The pros and cons of sex have been debated for a century, but that debate has previously not included MGEs as the likely original beneficiaries of DNA-damaging sex.

Our understanding of eukaryotic genomes is incomplete. What remains is to account for the vast regions of “single-copy” sequences that appear as unannotated blank lines on genomic maps: a task for the future.

## Supplementary Material

gkaf589_Supplemental_File

## Data Availability

No new data are presented in this article.
