# Supplementary material for: The biological and evolutionary consequences of competition between DNA sequences that benefit the cell and DNA sequences that benefit themselves
Source: Nucleic Acids Res. 2025 Jul 8;53(13):gkaf589. doi: 10.1093/nar/gkaf589 (PMC12235515; doi:10.1093/nar/gkaf589)
Supplement: gkaf589_Supplemental_File [file gkaf589_supplemental_file.pdf]

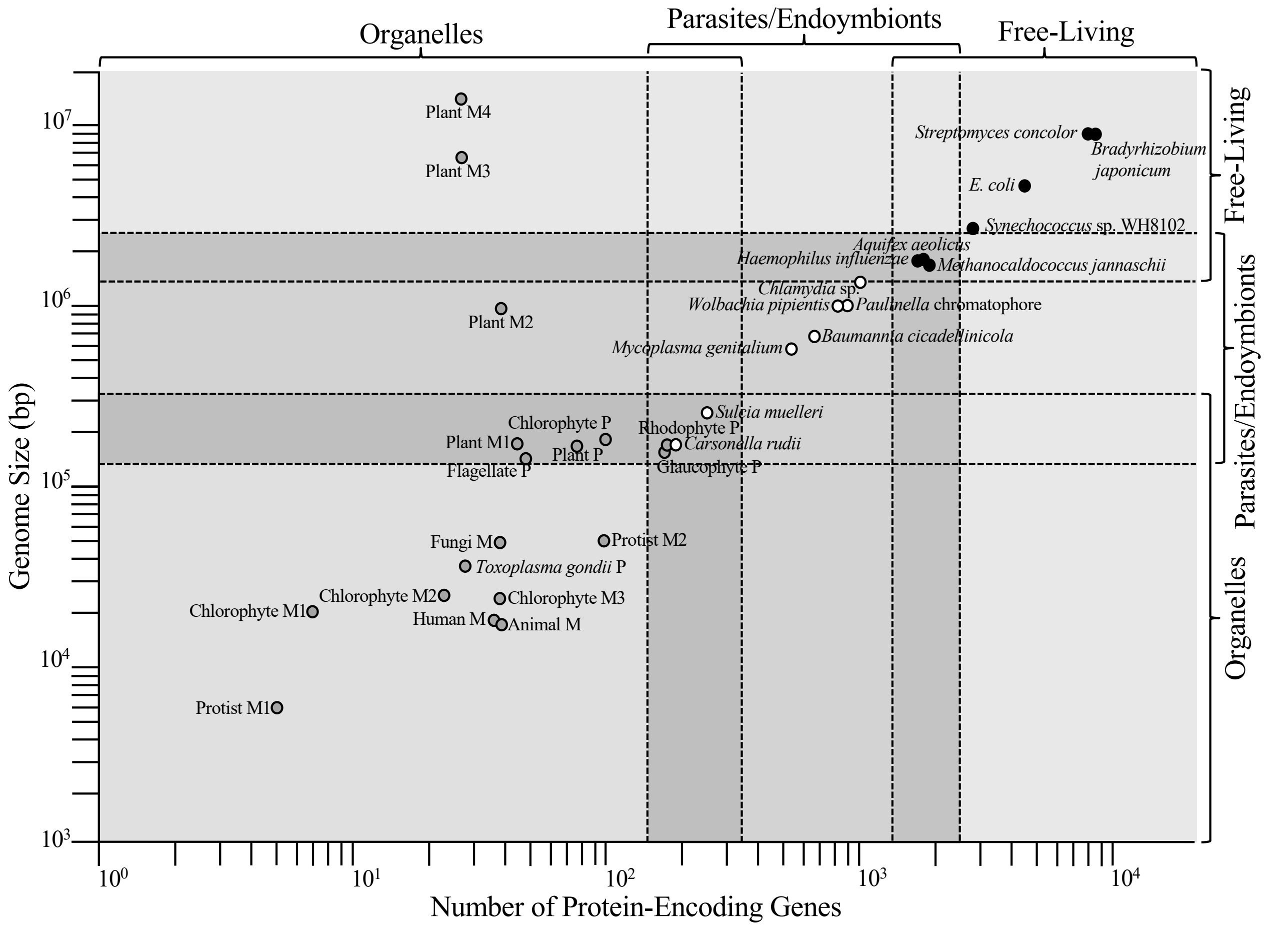

Supplementary Figure 1. Genome sizes and number of genes for free-living bacteria, parasites/endosymbionts, and organelles. All free-living organisms (black circles) have genome sizes greater than approximately 1.2 Mb and have at least 1200 genes. Parasites, commensals, and endosymbionts (white circles) have genome sizes between about 120 kb and 2.4 Mb, with 120 to 2300 genes. Most organelles (gray circles) have genome sizes less than 310 kb (except for some mitochondrial genomes), with fewer than 320 genes. Species names are provided for the first two categories, whereas general names for organelles are listed. P = plastid, M = mitochondrial. M1, M2, and M3 are chlorophytes with genomes of 20-25 kb, but 7-39 genes. Plant M1 is *Physcomitrella patens* (bryophyte). Plant M2 is *Cucurbita pepo* (angiosperm). Plant M3 is *Silene noctiflora* (angiosperm). Plant M4 is *Silene conica*. (Adapted from [15])

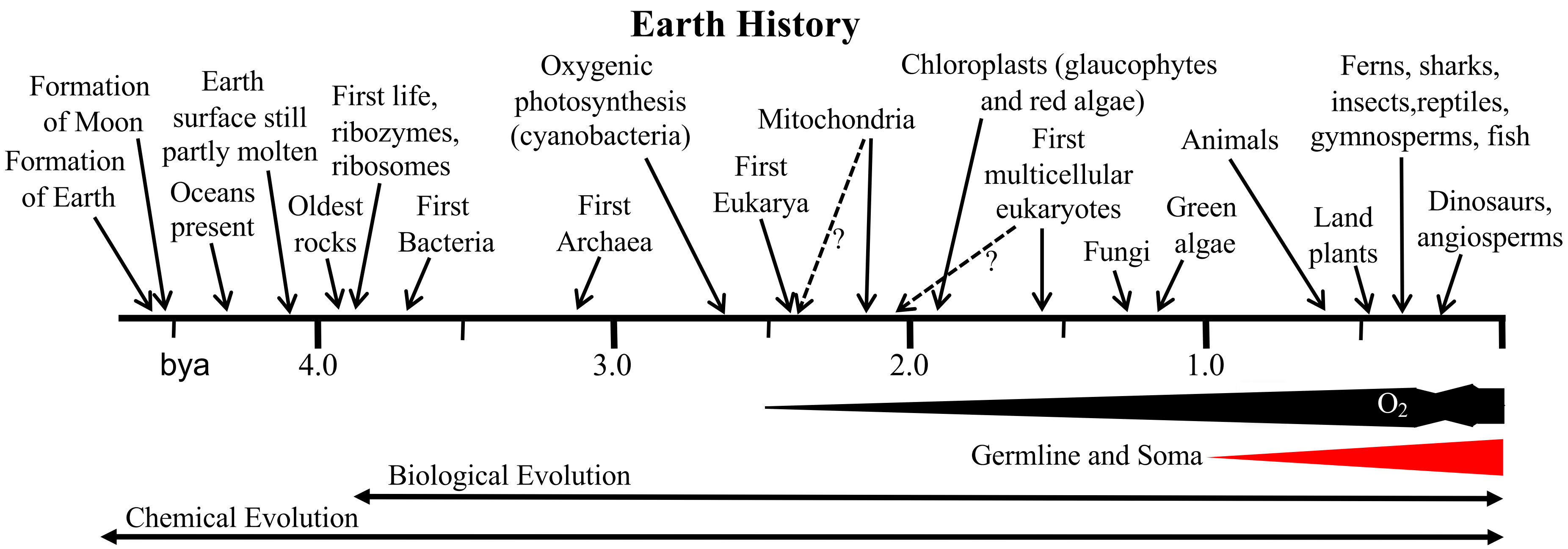

Supplementary Figure S2. Major events of Earth history (adapted from [15]). The Earth coalesced approximately 4.54 billion years ago. Since then it has undergone continued changes including changes in temperature, atmospheric conditions, asteroid impacts, worldwide glaciation events, and chemical and biological evolution, all of which continue today. Early events included collision of a Mars-sized body that led to the formation of the Moon, formation of oceans, vulcanism, and chemical evolution, including the evolution of RNAs, some of which were self-replicating and others that were enzymatic. Within 500 to 900 million years biological organisms appeared in a reducing atmosphere. As oxygen concentrations increased, aerobic organisms evolved, including eukaryotes with mitochondria. Another endosymbiotic event produced photosynthetic organisms with chloroplasts. Multicellular forms developed between 1.6 and 2.0 billion years ago, some of which developed sexual reproduction, followed by large increases in species diversity. Groups of multicellular organisms developed sexual reproduction, where there was a separation of a germline and a soma (somatic tissues; red triangle).

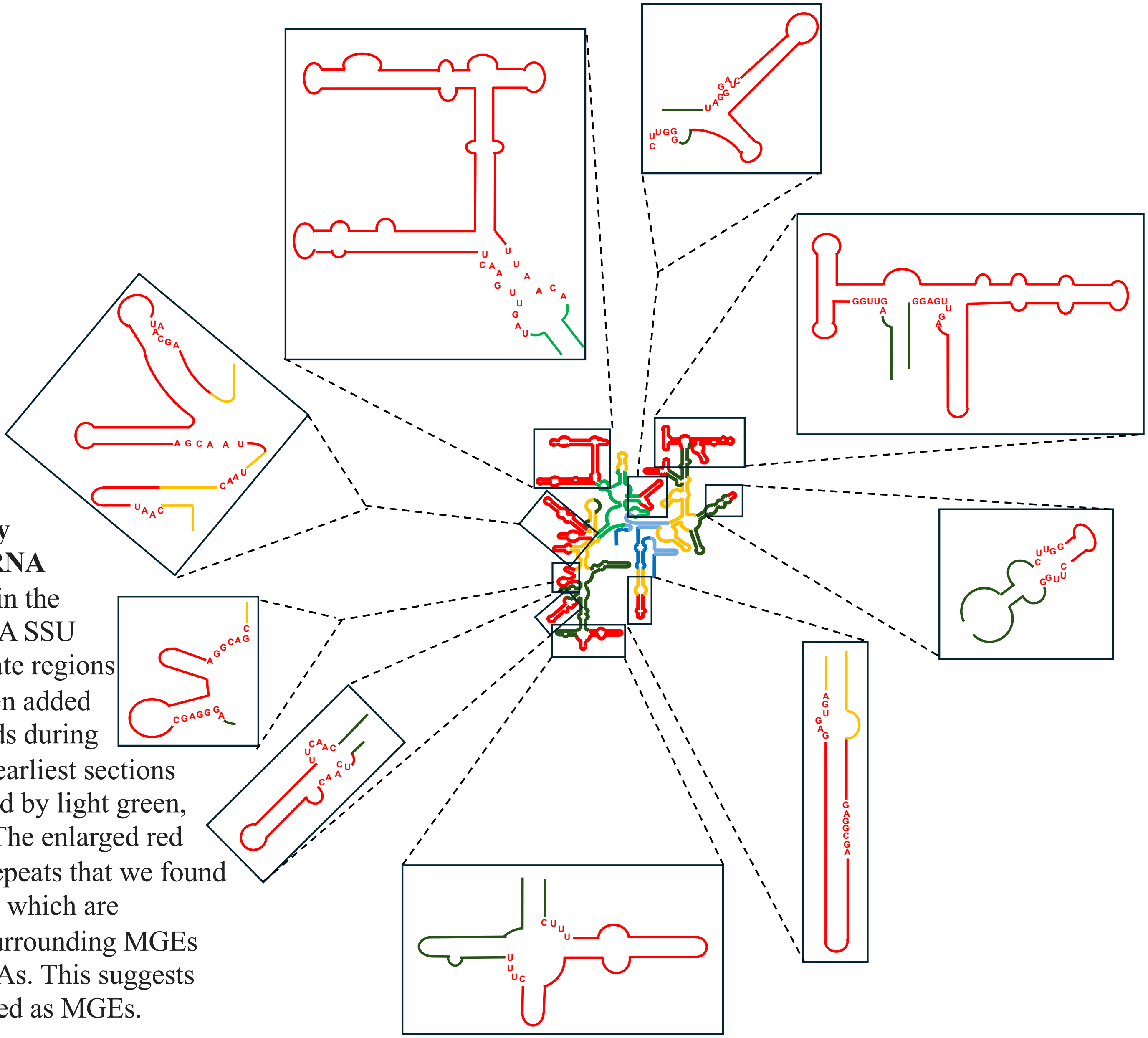

Supplementary Figure S3. Direct repeats in the vicinity of added segments of the rRNA small subunit. The diagram in the center is a model of the rDNA SSU [12, 29, 30] The colors indicate regions of the molecule that have been added during successive time periods during evolution of ribosomes. The earliest sections are indicated in blue, followed by light green, yellow, dark green, and red. The enlarged red regions highlight the direct repeats that we found at the borders of the sections, which are characteristic of sequences surrounding MGEs that have inserted within DNAs. This suggests that the red regions were added as MGEs.

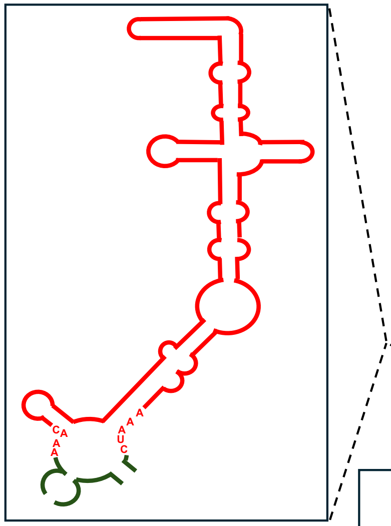

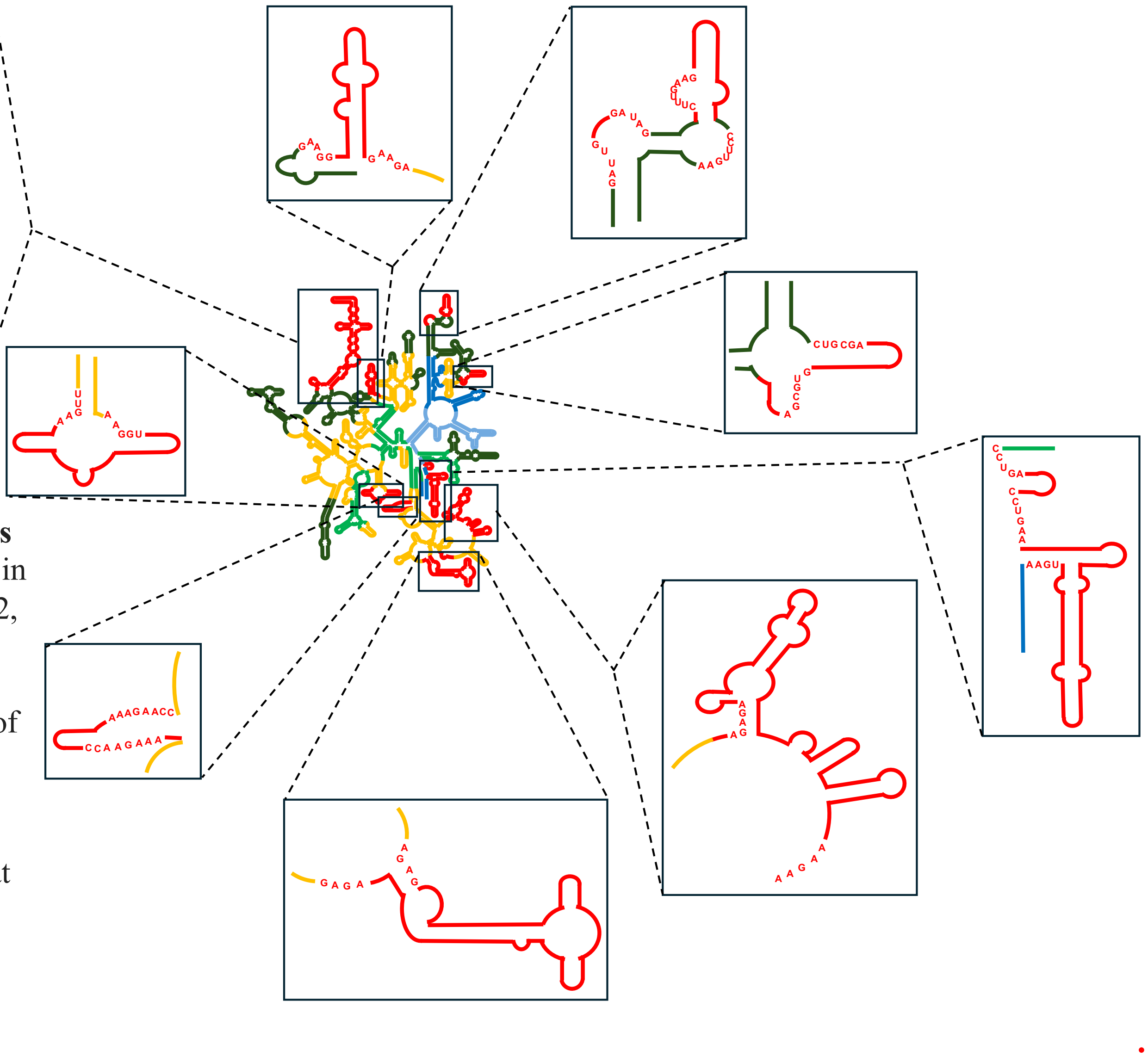

Supplementary Figure S4 Direct repeats in the vicinity of added segments of the rRNA large subunit. The diagram in the center is a model of the rDNA SSU [12, 29, 30]. The colors indicate regions of the molecule that have been added during successive time periods during evolution of ribosomes. The earliest sections are indicated in blue, followed by light green, yellow, dark green, and red. The enlarged red regions highlight the direct repeats that we found at the borders of the sections, which are characteristic of sequences surrounding MGEs that have inserted within DNAs. This suggests that the red regions were added as MGEs.

Supplementary Figure S5. Direct repeats in the vicinity of the joining of the acceptor arm and the TψC arm of tRNAs. The evolutionary pathway of tRNAs (top pathway) is based on Fujishima and Kanai (doi.org/10.3389/fgene.2014.00142); and Kanai (doi.org/10.3390/life5010321). The acceptor arm is the most ancient section of tRNA, followed by the TyC arm. We searched the border region and found two direct repeats (magnified in box) in those regions that could indicate the possible insertion of an MGE. This indicates that the TψC arm may have been an MGE that added to the acceptor arm. The acceptor arm attached to the TψC arm was used to produce a reverse complement strand that was ligated to the tRNA to essentially double the length of the tRNA to form the familiar cloverleaf structure of modern tRNAs.

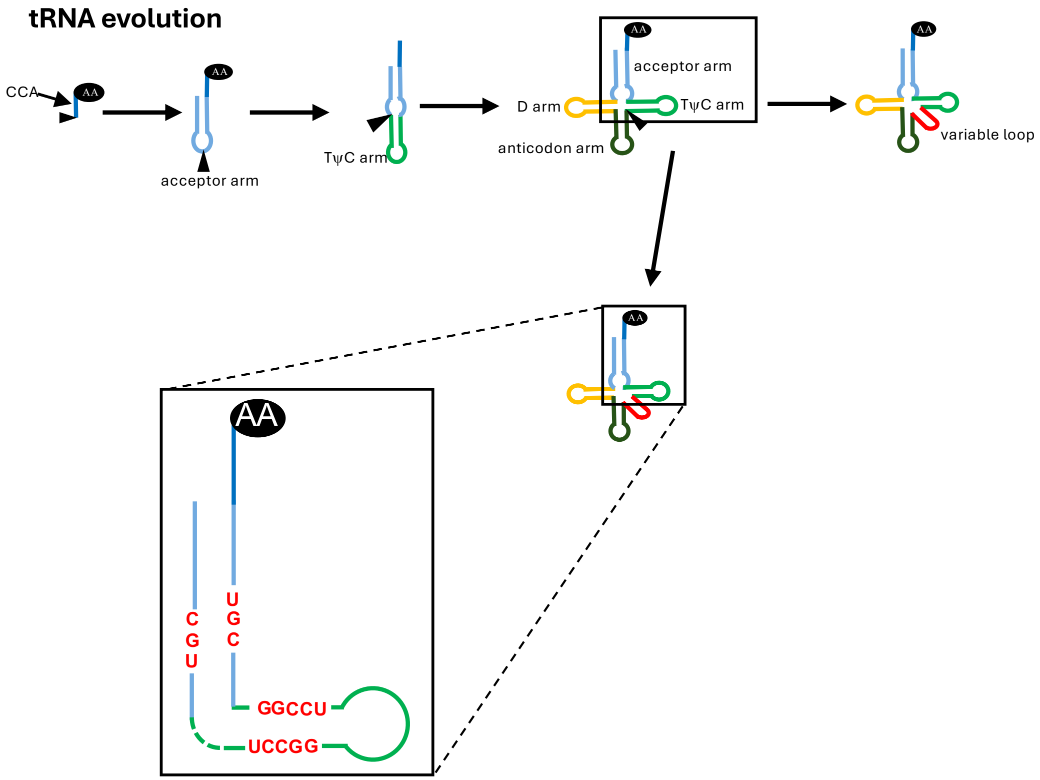

Supplementary Table S1. Genome sizes and number of genes for selected species, arranged in order of genome size (adapted from [15]).

Species	Taxonomic Group	Genome Size (kbp)	Number of Genes	Gene Density (genes per kb)[1]
RNA viruses (as a group)	RNA viruses	3.6 - 4.0	4 - 11	0.37 - 1.18
DNA viruses (as a group)	DNA viruses	5.0 - 1,200	11 - 1,260	0.47 - 2.03
Plastids (as a group)	Chloroplasts	40 - 1,350	26 -156	0.62 - 0.78
Mitochondria (as a group)	Mitochondria	1.0 - 12,000	1 - 90	0.0058 - 1.0
Carsonella ruddii (symbiont)	Bacterium	160	182	1.13
Nanoarchaeum equitans	Archaea	490	537	1.10
Mycoplasma genitalium	Bacterium	580	485	0.84
Methanococcus jannaschii	Archaea	1,700	1,780	1.05
Haemophilus infleunzae	Bacterium	1,800	1,740	0.97
Mycobacterium leprae	Bacterium	3,270	1,610	0.50
Mycobacterium lepromatosis	Bacterium	3,270	1,790	0.55
Mycobacterium tuberculosis	Bacterium	4,400	3,910	0.89
E. coli (K-12)	Bacterium	4,700	4,380	0.93
Methanosarcina acetivorans	Archaea	5,800	4,530	0.78
Cryptosporidium hominnis	Apicomplexan	10,400	3,990	0.38
Saccharomyces cerevisiae	Fungus	12,500	5,770	0.46
Sorangium cellulosum	Bacterium	14,800	11,600	0.89
Blastocystis subtype 7	Stramenopile	18,800	6,000	0.32
Plasmodium falciparum	Apicomplexan	23,000	5,270	0.23
Entamoeba histolytica	Amoebozoa	23,800	9,940	0.48
Plasmodium vivax	Apicomplexan	26,800	5,430	0.20
Echinamoeba silvestris	Amoebozoa	27,100	8,330	0.31
Dictyostelium discoideum	Amoebozoa	34,000	12,500	0.37
Trypanosoma cruzi	Excavata	34,000	22,600	0.66
Neurospora crassa	Fungus	39,000	10,100	0.26
Naeglaria gruberi	Excavata	41,000	15,700	0.38
Monosiga brevicollis	Opisthokonta	41,600	9,200	0.22
Acanthamoeba castellanii	Amoebozoa	46,000	16,800	0.37
Toxoplasma gondii	Apicomplexan	63,000	8,100	0.13
Paramecium tetreuelia	Ciliate	72,000	39,600	0.55
Caenorhabditis elegans	Nematode	97,000	19,000	0.20
Drosophila melanogaster	Insect	120,000	13,600	0.11
Arabidopsis thaliana	Plant	120,000	27,000	0.22
Phytophthora infestans	Stramenopile	240,000	17,800	0.074
Tetradon nigraviridis	Fish	340,000	27,900	0.082
Oryza sativa (rice)	Plant	390,000	28,200	0.072
Breviolum minutum	Alveolata	1,500,000	47,000	0.031
Euglena gracilis	Excavata	2,400,000	39,400	0.016
Zea mays (corn, maize)	Plant	2,400,000	59,000	0.025
Canis lupus familiaris (dog)	Mammal	2,500,000	20,000	0.0080
Mus musculus (mouse)	Mammal	2,500,000	22,000	0.0088
Homo sapiens (human)	Mammal	3,200,000	20,000	0.0063
Triticum aestivum	Plant	16,000,000	95,000	0.0059
Lepidosiren paradoxa	Fish	91,000,000	21,600	0.00024

[1]Parasitic bacteria (e.g., *Mycobacterium leprae, M. lepromatosis, Mycobacterium genitalium, M. tuberculosis*) have reduced functional proteome components, resulting in lower gene densities

(Supplementary Fig. S1; Silvia, F.J., et al. Microbiol. Spectrum, doi: 10.1128/spectrum.01692-21). However, they contain large numbers of pseudogenes and gene fragments. When those portions are included, gene densities are greater than 0.95 genes/kb. Mitochondrial and plastid genomes contain genes, pseudogenes, gene fragments, duplications, and unclassified sequences, resulting in genomes and proteomes that vary (sometimes greatly) from typical prokaryotic gene densities (Supplementary Fig. S1; Sloan, D.B., et al., 201x2, PLoS Biology, doi:10.1371/journal.pbio.1001241). Eukaryotic genomes also contain genes and pseudogenes, but they contain large proportions of MGEs and their derivatives, resulting in much lower gene densities.
